# Human T cells express CD25 and Foxp3 upon activation and exhibit effector/memory phenotypes without any regulatory/suppressor function

**DOI:** 10.1186/1479-5876-7-89

**Published:** 2009-10-22

**Authors:** Maciej Kmieciak, Madhu Gowda, Laura Graham, Kamar Godder, Harry D Bear, Francesco M Marincola, Masoud H Manjili

**Affiliations:** 1 Department of Microbiology & Immunology, Virginia Commonwealth University Massey Cancer Center, Richmond, USA; 2 Department of Pediatrics, Virginia Commonwealth University Massey Cancer Center, Richmond, USA; 3 Department of Surgery, Virginia Commonwealth University Massey Cancer Center, Richmond, USA; 4 Department of Pathology, Virginia Commonwealth University Massey Cancer Center, Richmond, USA; 5 Infectious Disease and Immunogenetics Section (IDIS), Department of Transfusion Medicine, Clinical Center and Center for Human Immunology (CHI), National Institutes of Health, Bethesda, USA

## Abstract

**Background:**

Foxp3 has been suggested to be a standard marker for murine Tregs whereas its role as marker for human Tregs is controversial. While some reports have shown that human Foxp3+ T cells had no regulatory function others have shown their role in the inhibition of T cell proliferation.

**Methods:**

T cell activation was performed by means of brayostatin-1/ionomycin (B/I), mixed lymphocyte reaction (MLR), and CD3/CD28 activation. T cell proliferation was performed using BrdU and CFSE staining. Flow cytometry was performed to determine Foxp3 expression, cell proliferation, viabilities and phenotype analyses of T cells.

**Results:**

Both CD4+ and CD8+ T cells expressed Foxp3 upon activation *in vitro*. Expression of Foxp3 remained more stable in CD4+CD25+ T cells compared to that in CD8+CD25+ T cells. The CD4+CD25+Foxp3+ T cells expressed CD44 and CD62L, showing their effector and memory phenotypes. Both FoxP3- responder T cells and CD4+FoxP3+ T cells underwent proliferation upon CD3/CD28 activation.

**Conclusion:**

Expression of Foxp3 does not necessarily convey regulatory function in human CD4+CD25+ T cells. Increased FoxP3 on CD44+ effector and CD44+CD62L+ memory T cells upon stimulation suggest the activation-induced regulation of FoxP3 expression.

## Background

In mice, scurfy mutation in forkhead/winged helix transcription factor gene *Foxp3 *causes autoimmune lesions including massive lymphoproliferation, diabetes, exfoliative dermatitis, thyroiditis and enteropathy. Such autoimmunity can be cured by a transgene encoding a wild-type *Foxp3 *allele [1]. The expression of Foxp3 in CD4+CD25+ T cells in wild-type mice and the diminished numbers of these T cells in scurfy and *Foxp3*-knockout (*Foxp3*^-^) mice suggested a role for Foxp3 in the development of regulatory T cells (Tregs) [2]. In addition, Foxp3 has been shown to be a specific marker for murine CD4+ Tregs because activation of non-T regs did not induce Foxp3 expression [2]. Ectopic expression of Foxp3 was shown to be sufficient to activate a program of suppressor function in peripheral murine CD4+ T cells [2].

In humans, the gene encoding Foxp3 was discovered during efforts to understand the genetic basis for a rare X-linked fatal autoimmune disease known as IPEX (immune dysregulation, polyendocrinopathy, enteropathy, X-linked) syndrome [3,4]. However, the role of Foxp3 as a key marker for Tregs in humans remains controversial. Unlike mice, activation of human CD4+ T cells by T-cell receptor (TcR) stimulation resulted in the expression of Foxp3 [5-12]. Most of these studies showed that induction of Foxp3, even in the presence of TGF-β, did not correlate with suppressive function of CD4+ T cells [6,10-12]. Although it was suggested that lack of suppression during the activation-induced expression of Foxp3 in human CD4+ T cells was because of transient expression of Foxp3, the observation still argues against a role for Foxp3 as key regulator of suppression in human CD4+ T cells upon expression. Regardless of the status of Foxp3, many studies considered CD4+CD25^high ^as Tregs in humans without being able to show their regulatory functions *in vivo *[13-15]. Most recently, it was reported that maternal alloantigens promoted development of Tregs in the human fetus that could suppress fetal antimaternal immunity. The authors considered CD4+CD25+Foxp3+ T cells as Tregs because of their partial suppressive function in a mixed lymphocyte reaction (MLR) *in vitro *[16]. These controversial reports prompted us to determine whether induction of Foxp3 expression in human T cells during activation and during MLR may confer regulatory functions. Our studies showed that activation-induced expression of Foxp3 was transient in CD8+CD25+ T cells but it was more stable in CD4+CD25+ T cells. These Foxp3+ T cells were mainly of effector and memory phenotypes.

## Methods

### Blood samples

PBMC were collected from two healthy donors, and duplicate experiments were performed.

### Flow cytometry

Three-color staining and FACS analyses were performed as previously described by our group [17]. Extracellular staining were performed using anti-human antibodies from Biolegend: PE- and FITC-CD25 (clone BC96), PE- and FITC-CD44 (clone IM7), FITC-CD62L (clone DREG-56), PE/Cy5-CD4 (clone OKT4) and PE/Cy5-CD8 (clone RPA-T8). Appropriate isotype control antibodies were used to exclude nonspecific binding. Foxp3 intracellular staining was done with PE anti-human Foxp3 Flow Kit (Biolegend, clone 206D) according to the manufacturer's protocol. Apoptosis was determined by staining of cells with Annexin V (BD Pharmingen).

### Proliferation assay

FITC BrdU Flow Kit (BD Pharmingen) was used in proliferation assays. T cells were also labeled with CFSE by incubation at 5 × 10^7 ^cells/mL in 5 μM CFSE/HBSS for 5 min at room temperature. Cells were then added with an equal volume of FBS, followed by three washes in FBS-containing HBSS.

### Mixed lymphocyte reaction (MLR)

Blood samples were diluted two-fold with PBS and layered onto Ficoll-Hypaque. Each tube was centrifuged at 400 g for 30 min and the lymphocytes at the interface were collected. These cells were washed once with RPMI 1640 medium containing 100 U/ml penicillin, 100 μg/ml streptomycin, and 2 mM L-glutamine. They were then resuspended at l0^7 ^cells/ml in the same medium containing 10% heat inactivated FBS. Allogeneic stimulating cells were irradiated in a cesium irradiator to a total dose of 5,000 rad, to abolish their capacity to proliferate. Cultures were set up in flat-bottomed 24-well plates and 3 × 10^6 ^responder cells were mixed with 2 × 10^6 ^irradiated stimulators in 2 mL. Cultures, set up in triplicates, were incubated for 8 days at 37°C. Control cells cultured with medium containing low dose IL-2 (20 U/mL) in order to maintain T cell viability during a 3-day culture. No IL-2 or anti-CD3 Ab was used in MLR samples. Some cultures were pulsed with 10 μM BrdU (BD Pharmingen).

### Statistical analysis

Statistical comparisons between groups were made using the Student *t *test with *P *< 0.0.5 being statistically significant.

## Results and discussion

### Activation of T cells induces expression of CD25 and Foxp3 associated with effector and memory phenotype differentiation

**Figure 1:**
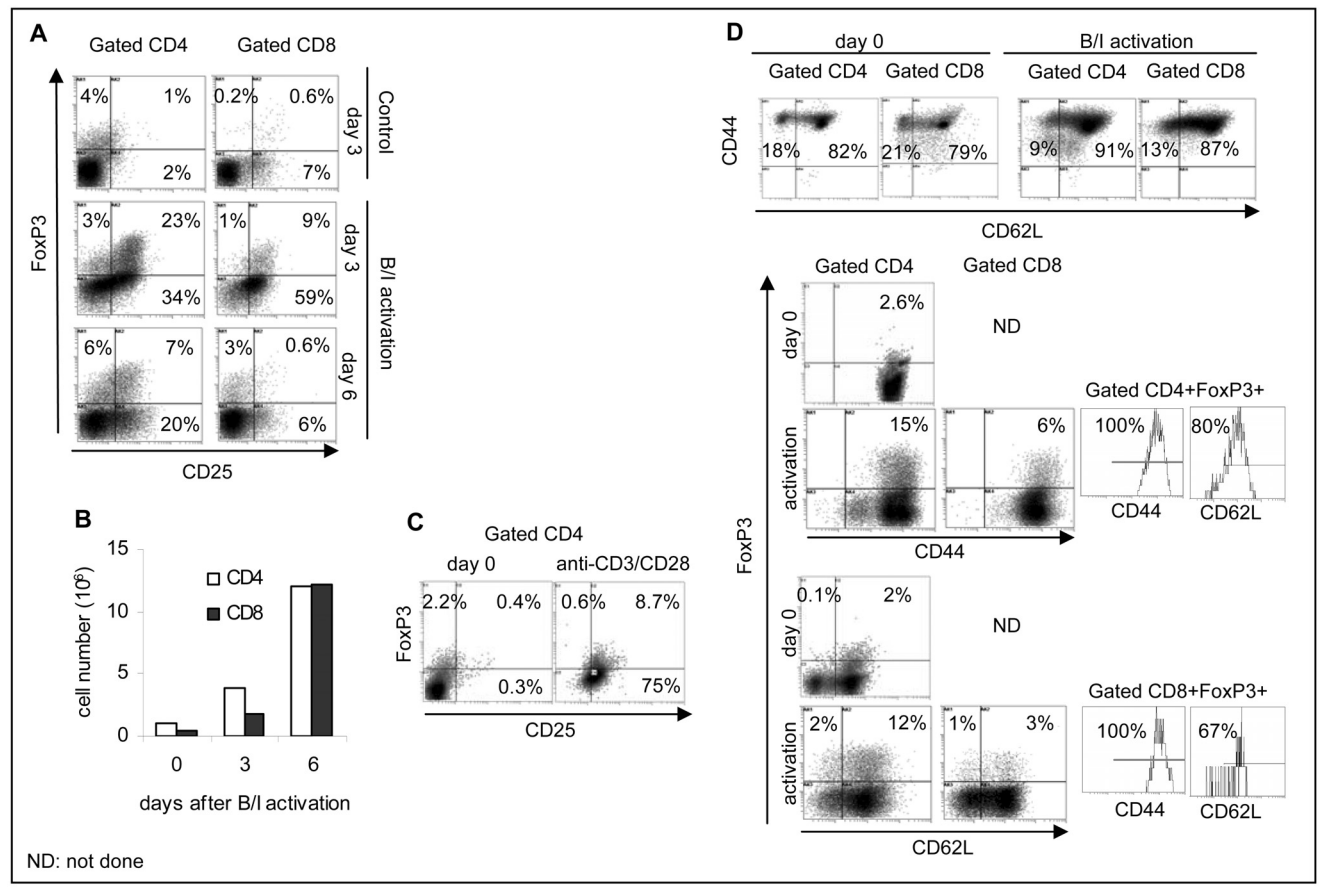
**Foxp3 expression following T cell activation**. T cells were isolated from healthy volunteers and split into two groups. Control group remained unactivated and cultured in the presence of IL-2 for 3 days (A; top panel) and another group was activated with B/I for 16 h and cultured in the presence of IL-2 for 3 days (A; middle panel) or 6 days (A; bottom panel). Absolute numbers of CD4+ and CD8+ T cells on pooled samples were determined on days 0, 3, and 6 post-culture by flow cytometry analysis (B). Expression of FoxP3 and CD25 were determined in freshly isolated CD4+ T cells (day 0) and after a 3-day stimulation with anti-CD3/CD28 Abs (C). Freshly isolated and B/I-activated T cells were subjected to flow cytometry to determine T cell phenotypes (D; top panel); Foxp3+ effector and memory T cells were determined in gated CD4+Foxp3+ cells or gated CD8+Foxp3+ cells (D; bottom panel). Representative data are shown from two donors in duplicate experiments.

PBMC were stimulated with bryostatin-1 (5 nM) and ionomycin (1 μM) (B/I) in the presence of 80 U/mL of IL-2 (Peprotech) for 16 h. B/I activation mimic intracellular signals that result in T cell activation by increasing protein kinase C activity and intracellular calcium, respectively [18-20]. Cells were washed three times and cultured at 10^6 ^cells/mL in complete medium with 40 U/mL IL-2 (Peprotech) for 3 days and expression of Foxp3 was determined using flow cytometry analysis. Expression of FoxP3 was also determined on freshly isolated T cells on day 0. As shown in Fig. 1A (top panel), presence of IL-2 alone for 3 days did not markedly increase expression of Foxp3 or CD25 above baseline levels on day 0 (Fig. 1C). The B/I activation, however, induced Foxp3 and CD25 expression in CD4+ and CD8+ T cells (Fig. 1A, middle panel). Upon B/I activation, CD4+CD25+Foxp3+ T cells were increased from 1% to 23% (*P *= 0.016) and CD8+CD25+Foxp3+ T cells were increased from 0.6% to 9% (*P *= 0.013). Extension of culture in the presence of IL-2 for 6 days without any further stimulation retained CD4+CD25+Foxp3+ T cells above the baseline levels in unactivated T cells (1% vs. 7%; *P *= 0.031) whereas CD8+CD25+Foxp3+ T cells dropped to baseline levels (0.6%). These results suggest that activation-induced expression of Foxp3 in CD4+CD25+ T cells is more stable than that in CD8+CD25+ T cells. Absolute number of T cells increased 3 and 6 days after the B/I stimulation and expansion in the presence of IL-2 (Fig. 1B). Activation of T cells by means of anti-CD3/CD28 Abs for 3 days produced similar results as for B/I activation by increasing CD4+CD25+FoxP3+ T cells from 0.4% to 8.7% (Fig. 1C). Phenotype analyses of T cells revealed CD44+ effector and CD44+CD62L+ memory phenotypes prior to and 6 days after the B/I activation (Fig. 1D, top panel). While effector CD4+ and CD8+ T cells were reduced after activation (18% to 9% and 21% to 13%, respectively), memory CD4+ and CD8+ T cells were increased (82% to 91% and 79% to 87%, respectively). Upon B/I activation, CD4+ T cells showed a 6-fold increases of FoxP3 expression in CD44+, CD62L+ phenotypes (CD44+: 2.6% to 15%; CD62L+: 2% to 12%). In addition, both CD4+ and CD8+ T cells showed FoxP3^high ^expression following activation compared to FoxP3^low ^expression on day 0 (Fig. 1D, middle and bottom panels). All CD4+Foxp3+ T cells expressed CD44 among which 80% also expressed CD62L (Fig. 1D, middle panel, far right). These data show that 20% of CD4+Foxp3+ T cells are effector and 80% are memory phenotypes. A similar phenotypic trend was detected for CD8+Foxp3+ T cells, showing 100% CD44+ of which 67% were CD62L+ T cells (Fig. 1D, bottom panel, far right). These results show that 33% of CD8+Foxp3+ T cells are effector and 67% are memory phenotypes. Data presented in Figs. 1A-D suggest that increased expression of FoxP3^high ^in effector T cells was due to the cell differentiation rather than cell proliferation, because relative percent of CD44+CD62L- effector T cells decreased after B/I activation. Similar mechanism may exist in memory T cells because of the expression of FoxP3^high ^after activation compared to FoxP3^low ^on day 0.

### Activation-induced FoxP3 expression in CD4+ T cells fails to convey regulatory function *in vitro*

**Figure 2:**
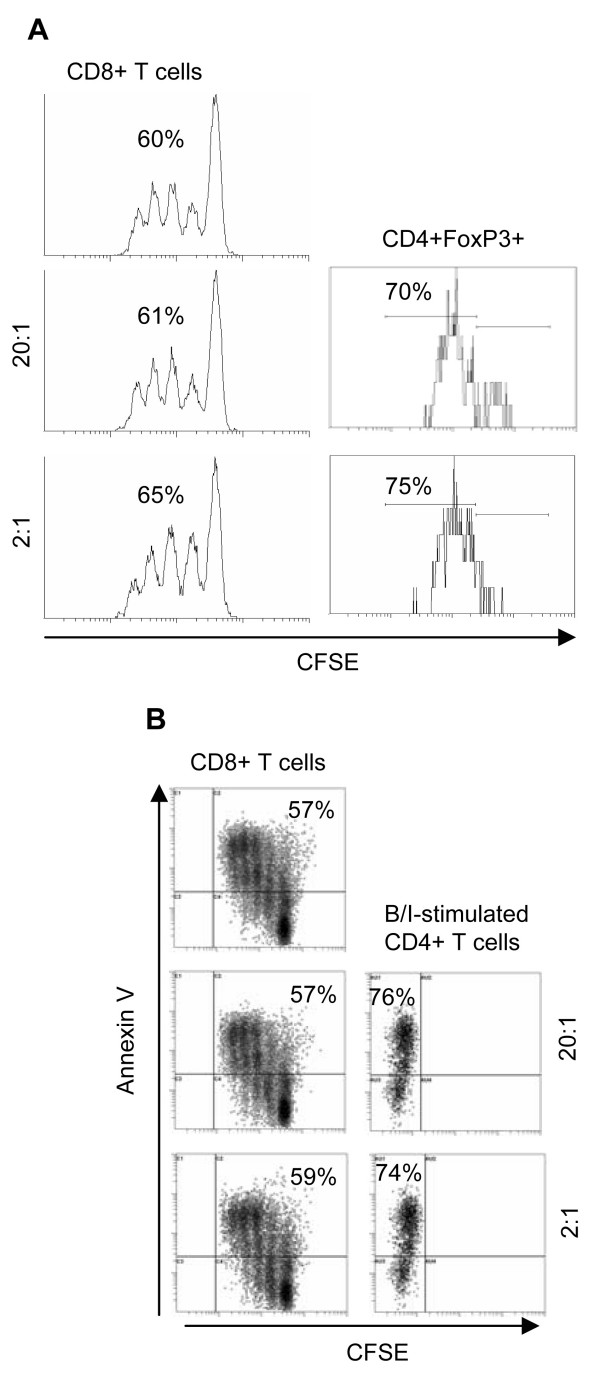
**T cell proliferation in the presence of inducible CD4+FoxP3+ T cells**. To perform a co-culture suppression assay, responder T cells were labeled with CFSE and cultured in the absence or presence of different ratios of inducible FoxP3+ T cells (20:1 and 2:1) for 3 days in the presence of anti-CD3/CD28 Abs. Gated CD8+ T cells showed CFSE dilution (A, left panel). Responder CD4+ T cells that expressed FoxP3 due to a 3-day activation were also gated and analyzed for CFSE dilution (A, right panel). Cells obtained from a co-culture suppression assay (A, left panel) were also stained for Annexin V in order to determine apoptosis in responder CD8+ T cells (B, left panel) and the B/I-activated CD4+FoxP3+ T cells (B, right panel).

T cells were labeled with CFSE and stimulated with anti-CD3 (1 ug/ml) and anti-CD28 (1 ug/ml) Abs in the presence or absence of the B/I-activated CD4+CD25+FoxP3+ T cells (2:1 and 20:1 responder:suppressor ratios) for 3 days. Flow cytometry analysis showed similar rates of proliferation of gated CD8+ T cells in the absence or presence of inducible FoxP3+ T cells (Fig. 2A, 60% vs. 61% and 65%). The CD3/CD28 activation also induced FoxP3 expression in responder CD4+ T cells. Gated CD4+FpxP3+ T cells also showed 70-75% proliferation upon activation (Fig. 2A). Analysis of T cell apoptosis revealed similar rates of apoptosis in responder T cells in the absence or presence of CD4+FoxP3+ T cells (Fig. 2B, 57% vs. 57 and 59%). Majority of the B/I-activated CD4+FoxP3+ T cells (74-76%) were found to be apoptotic during anti-CD3/CD28 activation in co-culture with responder T cells.

### Allogeneic activation of T cells during MLR induces Foxp3 expression in CD4+CD25+ T cells associated with effector/memory phenotype

**Figure 3:**
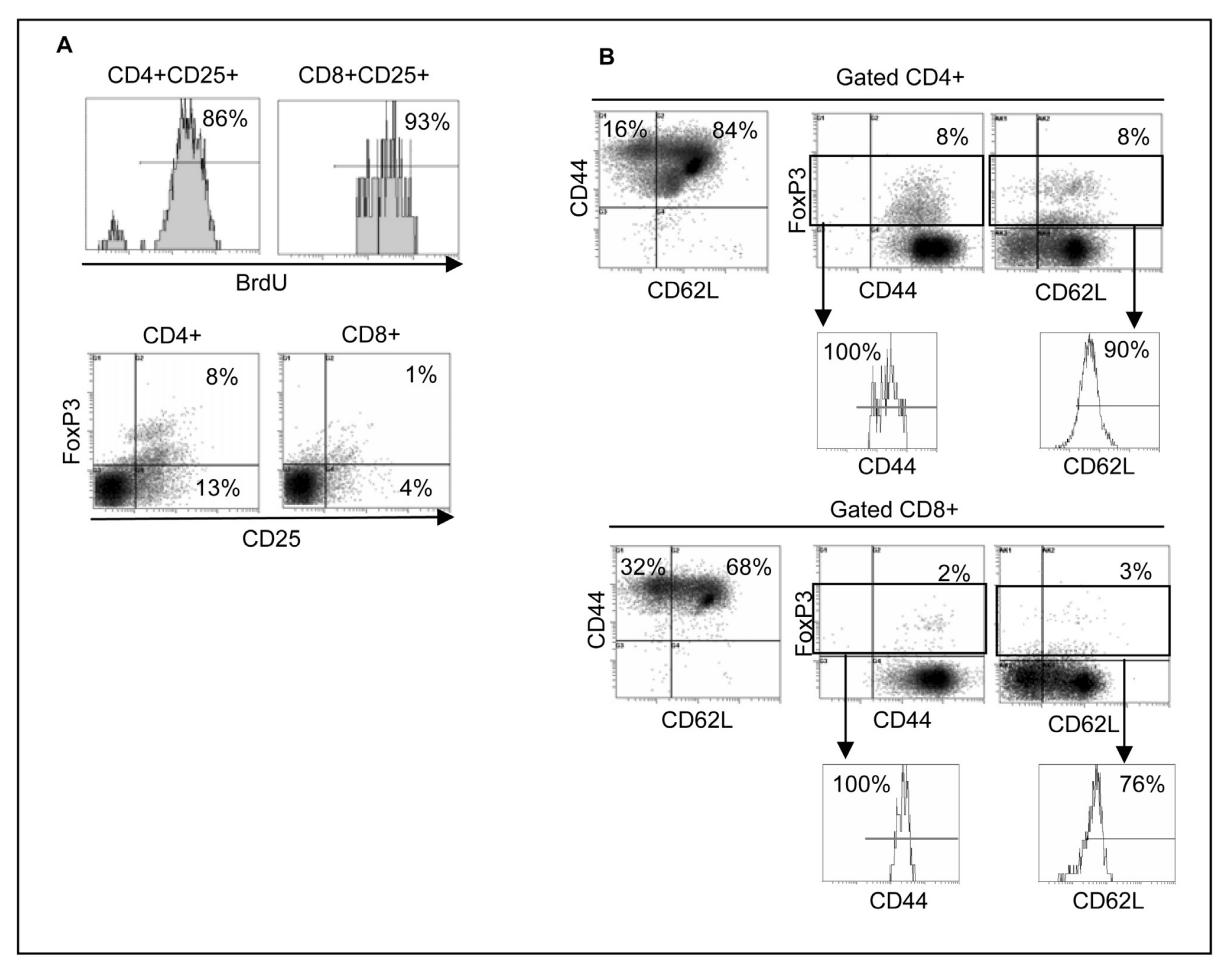
**Foxp3 expression following allogeneic MLR**. Cells were analyzed by flow cytometry after an 8-day MLR. BrdU incorporation was determined on gated CD4+CD25+ or CD8+CD25+ T cells (A; top panel). Gated CD4+ or CD8+ T cells were analyzed for the detection of CD25+Foxp3+ cells (A; bottom panel). Gated CD4+ T cells (B; top panel) or CD8+ T cells (B; bottom panel) were analyzed for the expression of CD44, CD62L, Foxp3. The CD44+ and CD62L+ T cells were determined by gating on CD4+Foxp3+ or CD8+Foxp3+ T cells. Representative data are shown from two donors in duplicate experiments.

We performed an 8-day allogenic MLR to determine whether induction of Foxp3 expression in T cells was stable during MLR and whether such an induced Foxp3+ expression might inhibit T cell proliferation. Responder and stimulator cells were obtained from different healthy donors. Stimulator cells were irradiated (5000 rad) and cultured with responder cells for 8 days in the presence of 10 μM BrdU (BD Pharmingen). Cells were then stained with relevant Abs and subjected to flow cytometry analysis. As shown in Fig. 3A (top panel) 86% of CD4+CD25+ T cells and 93% of CD8+CD25+ T cells showed BrdU incorporation as a result of cell proliferation. No proliferation was detected in the responder or stimulator cells alone (data not shown). Such allogenic proliferation took place in the presence of an activation-induced Foxp3 expression in CD4+ T cells such that 8% of CD4+ T cells were CD25+Foxp3+ (Fig. 3A, bottom panel). CD8+CD25+ T cells, on the other hand, did not show stable expression of Foxp3. These results are consistent with our observation in Fig. 1 showing that expression of Foxp3 in CD4+ T cells is more stable than that in CD8+ T cells 6-8 days following T cell activation. In previous reports, suppressive assays *in vitro *were conducted in the presence of high ratios of CD4+CD25+ T cells (Tregs) to responder cells, to determine the suppressive function on T cell activation and proliferation. Such artificial increases in the ratio of CD4+CD25+ T cells to responder cells would reduce *in vivo *validity of the observation. The frequency of CD4+CD25+Foxp3+ T cells induced during MLR was 8% which is considered to be within the physiologically relevant range as reported by other groups [21-24]. Frequency of naturally occurring Tregs in mouse is also around this range, yet having regulatory effects for the inhibition of autoimmunity. If Foxp3 expressing CD4+ T cells had any regulatory function, it should have inhibited cell proliferation during the culture *in vitro*. Similar to B/I-induced T cell activation, T cell phenotypes in a MLR included CD44+ effector (16%) and CD44+CD62L+ memory T cells (84%) (Fig. 3B). Again, all CD4+Foxp3+ T cells expressed CD44 among which 90% also expressed CD62L (Fig. 2B). These data show that 10% of CD4+Foxp3+ T cells are effector and 90% are memory phenotypes. A similar phenotypic trend was detected for CD8+Foxp3+ T cells, showing 100% CD44+ of which 76% were CD62L+ T cells. These results show that 24% of CD8+Foxp3+ T cells are effector and 76% are memory phenotypes. Lack of regulatory function in these Foxp3+ T cells may be because of their effector/memory phenotype since it has been reported that expression of Foxp3 in human memory T cells resulted in diminished suppressor activity [25]. In addition, Treg type 1 (Tr1) cells confer suppressor function in the absence of FoxP3 expression [26]. Given the role of Foxp3 as master regulator of Treg lineage commitment and maintenance in mouse [27], it does not seem to have such bona fide regulatory function for Treg lineage commitment in human T cells.

## Conclusion

In conclusion, the present study shows that Foxp3 expression is not a reliable marker for human Tregs. T cell activation, CD4+ T cells in particular, is associated with the expression of Foxp3 in effector/memory T cells without detectable regulatory function when present at physiologically relevant ratios.

## Abbreviations

PBMC: peripheral blood mononuclear cells; AICD: activation induced cell death; MLR: mixed lymphocyte reaction; T regs: regulatory T cells.

## Competing interests

The authors declare that they have no competing interests.

## Authors' contributions

MK performed B/I activation of T cells, flow cytometry, MLR, and BrdU proliferation assays; MG performed flow cytometry; LG performed B/I activation of T cells; KG participated in study design; HDB participated in study design and manuscript preparation; FMM participated in study design and data analysis; MHM designed the experiments, analyzed data, and prepared the manuscript.

All authors read and approved the final manuscript.
